# Measuring cognitive outcomes in a pre-clinical bioethics course

**DOI:** 10.1007/s40037-012-0014-3

**Published:** 2012-04-28

**Authors:** Ashley K. Fernandes, Nicole Borges, Heather Rodabaugh

**Affiliations:** Department of Community Health, Wright State University Boonshoft School of Medicine, 290J White Hall, Dayton, OH 45435-0001 USA

**Keywords:** Bioethics, Undergraduate medical education, Bloom’s Taxonomy of Learning

## Abstract

Medical schools universally accept the idea that bioethics courses are essential components of education, but few studies which measure outcomes (i.e., knowledge or retention) have demonstrated their educational value in the literature. The goal of this study was to examine whether core concepts of a pre-clinical bioethics course were learned and retained. Over the course of 2 years, a pre-test comprising 25 multiple-choice questions was administered to two classes (2008–2010) of first-year medical students prior to the start of a 15-week ethics course, and an identical post-test was administered at the end of the course. A total of 189 students participated. Paired *t* tests showed a significant difference between pre-test scores and post-test scores. The pre-test average score was 69.8 %, and the post-test average was 82.6 %, an increase of 12.9 % after the ethics course. The pre- and post-test results also suggested a shift in difficulty level of the questions, with students finding identical questions easier after the intervention. Given the increase in post-test scores after the 15-week intervention, the study suggests that core concepts in medical ethics were learned and retained. These results demonstrate that an introductory bioethics course can improve short-term outcomes in knowledge and comprehension, and should provide impetus to educators to demonstrate improved educational outcomes in ethics at higher levels of B.S. Bloom’s Taxonomy of Learning.

## Introduction

Medical schools in the United States universally accept the idea that a bioethics curriculum is an essential component of medical education, particularly in the pre-clinical years. All medical schools in the United States, for example, contain an ethics component within the first two years of medical school [1]. Surveys of ethics syllabi from medical schools in the United States and Canada have shown that bioethics courses focus on a discrete group of ‘core concepts’—the ‘nuts and bolts’ of a basic bioethics education [2, 3]. The hope among bioethics educators is that knowledge learned in these courses will one day influence the young physician’s behaviour and translate into ethical action. However, few studies [4] measuring outcomes such as learning and retention have demonstrated the educational value of a bioethics course in the pre-clinical years.

B. S. Bloom’s ‘Taxonomy of Learning’ [5] describes a pyramid of educational goals which can be applied to course design; as one ‘moves up’ the pyramid, teaching and evaluation become more complex. The use of Bloom’s pyramid in curricular design has been employed in evaluating basic science knowledge, but has not yet been utilized in the medical humanities [6]. This project chose to focus on the most basic levels of Bloom’s pyramid—knowledge and comprehension—to determine whether administration of a pre- and post-test could demonstrate cognitive gains in understanding core concepts in bioethics after a 15-week course. As one ‘moves up’ the pyramid (toward Bloom’s educational zenith of synthesis and evaluation), teaching and learning become more complex, but prior to reaching the summit, the foundation must be firmly established. This is particularly true in bioethics education, where educators hope to instil foundational knowledge which ultimately changes behaviour. But we currently lack even the antecedent data to answer the question: do medical students know the basics of bioethics?

The purpose of the initial phase of this study was to determine whether the core concepts of a pre-clinical medical ethics course are learned and retained as measured by the administration of pre- and post-test multiple-choice questions. The hypothesis was that short-term learning and retention in bioethics would occur. Support of this hypothesis would justify further research into long-term retention and reinforcement of concepts considered to be of importance in a comprehensive medical ethics education.

## Method

This study was approved as exempt by the Wright State University School of Medicine Institutional Review Board. Core concepts in bioethics were identified through a literature review of the syllabi content of ethics classes in medical schools, and 30 core concepts were identified (Table 1) [1–3].

**Table 1:** Core concepts in bioethics in the US and Canadian medical schools [1–3]

Abortion	Brain death/coma	Economics/health care delivery	Geriatrics	Medical student ethics	Religion/spirituality
Access to care	Competence	Ethical theories	Global/public health	Organ transplantation	Resource allocation
Advance directives	Confidentiality	Ethics committees	Informed consent	Palliative care/hospice	Reproductive technologies
Autonomy	Conflicts of interest	Euthanasia/assisted suicide	Legal issues in medicine	Paediatric ethics	Truth-telling
Bioterrorism	Disabilities	Genetic testing	Malpractice/medical errors	Professionalism	Women’s health

From the list of core concepts, learning objectives for each question were generated, and a multiple-choice question bank was developed by two bioethics experts (AKF and MTW) using appropriate guidelines to ensure content validity [7]. Since there was no prior validated bioethics exam available, a pre-test of 25 multiple-choice questions was then created. First-year medical students were given a unique identification number which they used as a login to the ethics testing website (i.e., WebCT), and which will preserve anonymity for future studies.

During the academic years 2008–2010, a pre-test comprising 25 multiple-choice questions was administered to 197 students (post-test *n* = 189; 96 % total response rate) prior to the start of the first-year ethics course. The students had no prior ethics training in medical school. The course consisted of fifteen 3-h sessions, typically with a 1–2 h panel or presentation, followed by small-group sessions. The small groups, comprising 11–12 students, were facilitated by 1–2 trained faculty members and explored the ethical topic in greater depth. A post-test containing identical questions was administered six months after the pre-test, at the end of the 15-week course.

## Results

Over two years, results of the pre-test showed a percentage score of 69.8%. On the post-test, the percentage score was 82.6%, which is an increase of 12.9 % after completing the 15-week course. Analysis using paired *t*-tests showed a significant difference (*p* < 0.05; Boneferroni correction *p* < .002) between pre-test raw scores (mean = 17.44, SD = 2.21) and post-test raw scores (mean = 20.66, SD = 2.39); *t*(188) = −16.16, *p* = < 0.001. Individual test questions were then analyzed for validity using both the discrimination factor and difficulty index [8]. Our discrimination factors (DFs) ranged from 0.44 to −0.05. The vast majority (72 %) had DFs in the acceptable range (>0.2) in pre- and post-testing. Only 3 of the 25 questions had negative discrimination factors (in the post-test only), suggesting lower performers scored very well on these questions only after the intervention. The difficulty index categories—whether an individual question was ‘challenging’ (mean score of <70 %); ‘moderate,’ (mean score of ≥70 to <90 %); or ‘easy’ (mean score of ≥90 %), were created using years of basic science test performance data at our institution to design exams for which a mean score would be 78–83 % and the failure rate would be <10 %. After analysis of the questions, the pre- and post-test results also suggested a shift in difficulty level of the questions, with students finding identical questions easier after the intervention. In both years of the study, 40 % of pre-test questions were considered ‘challenging’ to the students. After the ethics course was completed, the number of ‘challenging’ questions decreased to 16 and 8 % in year one and year two, respectively; the same pre-test questions shifted into the ‘moderate’ or ‘easy’ categories in the post-test. These findings suggest that true cognitive gains took place during the bioethics course.

## Conclusions

Given the increase in post-test scores after the 15-week interventions in two years, the study suggests that core concepts in medical ethics were learned and retained. While the pre-test mean of almost 70 % appears high, particularly in European educational systems, at our institution (and many American medical schools), a 60 % would be a failing score, and a 70 % requires course remediation. Therefore, an increase of 13 % in the mean after the course is both educationally and statistically significant. Whether such an increase is predictive of future changes in behaviour remains to be seen. But since we are only at the starting point on Bloom’s pyramid, the answer to that profound query is beyond the scope of our study. From an educator’s standpoint, most would not be satisfied if students left medical school knowing only 70 % of the ‘core concepts’ of our subject matter. Thus, results demonstrating that an introductory bioethics course can improve short-term outcomes in knowledge and comprehension are gratifying. The study begins to answer the question of whether a bioethics course makes a difference in students’ technical knowledge.

Several limitations need to be considered in light of this study’s findings. First, the pre- and post-test questions were identical, which introduces the possibility that student recall may account for the improvement in post-test scores. Pretest sensitization, potentially leading to recall bias, has been an inherent problem in pre- and post-test study design. However, the literature [9, 10] suggests that our pre- to post-test interval of 6 months will reduce the likelihood of such bias.

A second limitation during the first year is that the students had a brief window of time (approximately one week) in which they could review annotated answers to the pre-test questions online. It is possible—despite the fact that they were instructed not to study for the post-test—that some students may have printed copies of the pre-test and used it as a study guide. It is not clear how many (if any) students did so. Motivation to ‘study’ was reduced by the fact that the pre- and post-tests did not count toward the students’ final grades, and that the students were explicitly asked not to prepare for the post-test. To address this concern, students in the second year had no access to the pre-test before the post-test. Neither group of students was aware that the questions would be identical to the pre-test.

Third, the pre- and post-test did not count toward the student’s final grade. While this fact helps to reduce the impact of the second limitation, it introduces the possibility that students may have (particularly on the post-test) not taken the test seriously. However, this limitation, if truly problematic, would likely serve to underestimate (rather than overestimate) the effectiveness of the intervention. In the second year of the study, students again were instructed not to study for the post-test. However, to address the concern that students would not take the post-test seriously, they were required to pass the test by earning a 70 %. While the grade itself did not factor into their grade for the course, the fact that they passed the test did.

## Future directions

The process of extending the study to test long-term retention of core concepts throughout all four years of medical school will occur in 2012–2013. The cohort tested in the current study will be re-tested at the end of the third and fourth years of medical school. The hypothesis is that cognitive gains in bioethics knowledge will be eroded over the course of four years of medical school. If accurate, the extension of the study will also serve as a needs assessment for curricular change, focusing on reinforcement and long-term retention of basic knowledge in ethics. If the basic knowledge bioethics educators wish to impart diminishes over time, curricular strategies might employ greater emphasis on behavioural and attitudinal modification (e.g., through the use of decision-oriented clinical cases or reflection essays during the clinical years) which re-emphasize core concepts and through better integration of bioethics throughout a four-year curriculum.
